# Assessing the Knowledge of Students about Dental Bleaching in Shahid Bahonar University of Kerman, Iran

**DOI:** 10.30476/DENTJODS.2020.86318.1183

**Published:** 2021-06

**Authors:** Marzieh Karimi-Afshar, Ali Eskandarizadeh, Molouk Torabi-Parizi, Reyhaneh Aftabi

**Affiliations:** 1 Dept. of Orthodontics, School of Dentistry, Kerman University of Medical Sciences, Kerman, Iran; 2 Dept. of Operative Dentistry, School of Dentistry, Kerman University of Medical Sciences, Kerman, Iran; 3 Social Determinants on Oral Health Research Center, Kerman University of Medical Sciences, Kerman, Iran; 4 Postgraduate Student of Pediatric Dentistry, School of Dentistry, Kerman University of Medical Sciences, Kerman, Iran

**Keywords:** Knowledge, Tendency, Bleaching, Students

## Abstract

**Statement of the Problem:**

An attractive smile and white teeth give self-confidence and provide impression of health, which help individuals for social and interpersonal success. Increasing demand for tooth bleaching and lack of relevant information on the other hand, necessitate a new investigation to assess the knowledge of the students about dental bleaching in Kerman.

**Purpose:**

This study was designed to assess the overall knowledge of the students of Shahid Bahonar University of Kerman regarding dental bleaching and their tendency to perform it.

**Materials and Method:**

This cross-sectional study enrolled 384 students who were selected by simple random sampling. A valid and reliable self-administered researcher-made questionnaire was employed to collect data about demographic information, health behavior, tooth bleaching, and the tendency to perform . This tool contained 8 questions with the focus on the knowledge of dental bleaching. Data were analyzed by using SPSS version 21 and regression analysis at a significant level of 0.05.

**Results:**

Overall, 59.55% of participants were male and 40.45% were female students. Most likely, the source for gaining information was internet. In this regard, 29 individuals were dissatisfied with tooth color, 27 ones with the appearance of teeth, 13.5% have done tooth bleaching, and 69% had tendency to do that. The preferred knowledge was about the difference between scaling and root planning (SRP) and bleaching. There was no significant difference between age, gender, and marital status variables with the knowledge of dental bleaching. Tooth color satisfaction increased the tendency to do bleaching about 1.87 times.

**Conclusion:**

The study highlights that 69 % of the students had a tendency to do the bleaching, their knowledge was moderate, and there was no statistical relationship between knowledge, gender, and marital variables. Color variable had an overall positive effect on the tendency to do the bleaching.

## Introduction

People's appearance plays a key role in their social interactions; subsequently, the color of teeth is the most important determinant of patient satisfaction [ 1]. Recently, dental procedures have undergone changes especially in Western societies, because dental caries have been decreased, the numbers of remaining teeth have been increased, and the patients' demand for cosmetic dentistry is increasing [ 2- 3]. The first report on tooth bleaching is cited in Zakaria Razi's Book of Al-Mansouri [ 4]. Feinman [ 5] developed the first bleaching method, using hot oxygenated water. Tooth bleaching in Iran started in 1335 at the School of Medicine and since then has been practiced by the Iranian dental schools [ 6].

Bleaching can be done with the use of carbamide peroxide, hydrogen peroxide, and sodium perborate with or without light activation. There are various techniques of vital bleaching, including bleaching in the office by high concentrations of bleaching materials with soft tissue protection or at-home bleaching by lower concentrations of bleaching materials [ 7- 9]. Some negative effects such as nausea, tooth sensitivity, and gingivitis have been reported with the procedure [ 10- 13].

Azodo and Ogbomo [ 14] and Mehl *et al*. [ 15] have reported that the arrangement and the size of teeth were important determinants of dental satisfaction for the Benin university students. Maghaireh *et al*. [ 16] found that 69.3% of patients were satisfied with the appearance of their teeth. However, Akarslan *et al*. [ 17] showed that among the patients referred to the dental School in Turkey, 55.1% were dissatisfied with tooth color and 42.7% with tooth appearance, while 29.9% of the patients were dissatisfied with tooth crowding and 23.3% of people concealed their teeth while smiling. Samorodnitzky-Naveh *et al*. [ 18] highlighted that the main factor in tooth beauty was tooth color, although 37.3% of the patients were not satisfied with the appearance of their teeth. According to Subait *et al*. [ 19] among 238 students in Saudi Arabia, 15% of the students were dissatisfied with tooth appearance, 62% with tooth color, 62% of them had tendency to bleaching and 32% wanted to have orthodontic treatment.

Increasing demand for tooth bleaching and lack of a similar study in Kerman provide a new investigation to assess the knowledge of the students about dental bleaching in Shahid Bahonar University of Kerman.

## Materials and Method

This descriptive-analytical cross-sectional study was conducted on the students of electrical, civil, industrial, chemistry, mechanics, metallurgy, computer and mining engineering, at Shahid Bahonar University of Kerman.

Data collection tool was a questionnaire that consisted of demographic information (gender, age, field of study, brushing frequency, dental flossing, and dental visits), some questions about tooth color satisfaction, importance of teeth appearance, tendency to do bleaching, history of dental bleaching and satisfaction, source of information, and eight questions about the knowledge of dental bleaching. The reliability and validity coefficient of the questionnaire were 0.81 and 0.79, respectively, using ICC formula. Sample size was based on the below formula: n= (z^2*p(1-p)) /d^2, considering z= 1.96, d= 0.05 and *p*= 0.5, was calculated to be 400, but some questions had been missed by the respondents, thus final analysis was performed on only 384 questionnaires. After collecting the questionnaires, the data were coded and entered into SPSS statistical software version 21, using descriptive statistics tests (Mean, Standard deviation, frequency, and percentage of frequency). The independent t-test, chi-square and logistic regression test was performed at significant level 0.05.

The participants were assured that they would not be named in any part of the research and the project would be completely voluntary and with their consent.

 This proposal is registered under the IR.KMU.REC. (1397, 441) and was approved by Kerman University of Medical Sciences Ethics Committee.

## Results

In this study, 59.55% of participants were male and 40.45% were female students (Table 1). In terms of oral hygiene behavior, 49.8% individuals brushed twice daily, 47% used dental floss and 35.3% had dental visits less than 6 months ago. In addition, 17.5% of the participants were reported to have smoking habit and 13, 5% were consuming hookah (Table 2).

**Table1:** Sample distribution according to demographic information

Variables		Frequency	%
Gender	Female	161	40.45
	Male	237	59.55
Marital status	Single	342	88.14
	Married	46	11.86
Field of study	Electrical engineering	50	12.5
	Chemistry engineering	50	12.5
	Industrial engineering	50	12.5
	Civil engineering	50	12.5
	Computer engineering	50	12.5
	Metallurgy engineering	50	12.5
	Mining engineering	50	12.5
	Mechanical engineering	50	12.5

**Table2:** Frequency distribution of individuals according to oral health behavior

Variable		Number	Percent
Tooth brush behavior (396) using floss	Once in a day	57	14.3
	Two times a day	199	49.8
	More than two times a day	113	28.3
	Seldom	27	6.8
	Yes	188	47
	No	124	31
	Seldom	88	22
Last time of dental visit (394)	Less than 6 months ago	141	53.3
	6 months ago	35	8.8
	One year ago	59	14.8
	Two years ago	53	13.3
	Do not remember	106	26.5
Cigarette smoking (395)	No	326	81.3
	Yes	70	17.5
Hookah smoking (398)	No	270	67.3
	Yes	129	32.2
Cigarette smoking at the present (401)	No	371	92.5
	Yes	30	7.5
Hookah smoking at the present (396)	No	342	85.3
	Yes	54	13.5

Overall, 87% of people considered the appearance of teeth important and very important, whereas, 26.75% of the participants were dissatisfied and completely dissatisfied with the color of their teeth. 

Only 44.33% of participants that had access to Internet presented more information on tooth bleaching and 14.09% gained their information by consulting their dentist. Moreover, 13.5% of the participants did bleaching and 69% (42.33% of females and 57.97% of males) tended to do it. The highest tendency (47.41%) for bleaching was in dental office and the least favorable method was the use of bleaching tapes (1.44%). The most correct answer was about the difference between bleaching and scaling and root planning (SRP) (52.1%). 

By using univariate and multivariate analysis, 17.88% of female and 12% of male students have done bleaching treatment, which was not statistically significant (*p*< 0.111). The percentage of students who did bleaching was not different between married and single ones (*p*= 0.771), although female students heard more about dental bleaching (*p*< 0.001). There was no statistically significant difference in the number of participants performed bleaching in different fields. There was a significant difference, regarding the dissatisfaction of tooth color in the students with a tendency to do bleaching (76.21%) and those without a tendency to do bleaching (2.1%), (*p*<0.001). The mean score of knowledge and health behavior were higher in the students who heard about bleaching (*p*< 0.001, *p*< 0.001) and have done bleaching (*p*= 0.030, *p*< 0.012). 

Statistically, there was a significant difference between the amount of hygienic behavior and gender. Female students had significantly better hygienic behavior (*p*= 0.007). There was no significant difference (*p*= 0.000) between other variables including gender, field of study, and marital status (Table 3).

**Table3:** Realtionship between knowledge and attitude with demographic variables

Demographic variables		Have you ever heard anything about bleaching?		*p* Value	Have you ever done dental bleaching?		*p* Value	Do you have tendency to do bleaching?		*p* Value	Yes	No	Yes	No	Yes	No
Gender	Female	118(50.64%)	39(25.49%)	0.000	27(50%)	124(38.51%)	0.111	116(42.03%)	35(36.08%)	0.305
	Male	115(49.36%)	114(74.51%)		27(50%)	198(61.49%)		160(57.97%)	62(63.92%)	
Marital status	Single	200(87.72%)	131(87.92%)	0.945	44(81.48%)	279(89.14%)	0.110	236(87.41%)	85(88.54%)	0.771
Married		28(12.28%)	18(12.08%)		10(18.52%)	34(10.86%)		34(12.59%)	11(11.46%)	
Field of study	Electrical engineering	31(13.3%)	19(12.34%)	0.149	6(11.11%)	41(12.65%)	0.474	30(10.83%)	19(19.39%)	0.120
	Chemistry engineering	26(11.16%)	22(14.29%)		6(11.11%)	39(12.04%)		32(11.55%)	14(14.29%)	
	Industrial engineering	38(16.31%)	12(7.79%)		8(14.81%)	40(12.35%)		38(13.72%)	10(10.2%)	
	Civil engineering	24(10.3%)	22(14.29%)		3(5.56%)	44(13.58%)		34(12.27%)	12(12.24%)	
	Computer engineering	31(13.3%)	17(11.04%)		11(20.37%)	36(11.11%)		35(12.64%)	11(11.22%)	
	Metallurgy engineering	30(12.88%)	19(12.34%)		8(14.81%)	42(12.96%)		38(13.72%)	11(11.22%)	
	Mining engineering	30(12.88%)	18(11.69%)		7(12.96%)	40(12.35%)		41(14.8%)	6(6.12%)	
	Mechanical engineering	23(9.87%)	25(16.23%)		5(9.26%)	42(12.96%)		29(10.47%)	15(15.31%)	

Logistic regression test showed that among all the variables, the only variable that had significant statistical relationship with one's tendency to do bleaching was tooth color dissatisfaction, which has increased for 1.87 times.

## Discussion

This survey and the study of Dozic *et al*. [ 20] showed that the desire to have an attractive smile and white teeth, have currently become an important need. In the current study, 87% of people considered the appearance of teeth important and very important. Only, 26.75% of the participants were dissatisfied and completely dissatisfied with the color of their teeth, which is less than 76.4 % reported by Nomay [ 21].

The satisfaction rates of teeth color were reported to be 66.3% by Maghaireh *et al*. [ 16], 36.6% by Lanjert *et al*. [ 22], 43.1% by Al-Zarea *et al*. [ 23], 42.8% by Yu *et al*. [ 24], 32% by Nomay [ 21], and 26.6% by Subait *et al*. [ 19]. Their reported results were lower than the results yielded by our study (87 %), even though the tooth color may be the primary cause of dissatisfaction with tooth appearance [ 17]. 

In the current study, there was no statistically significant relationship between variables including gender, field of study, and age with dental color satisfaction. According to Vallittu *et al*. [ 25], color satisfaction has been shown to be age-dependent. The results of the study by Akarslan *et al*. [ 17] indicated that people with higher degrees of education and higher self-esteem were more satisfied with the color of their teeth. The reason for the lack of statistical correlation between color satisfaction with age and education in this investigation is probably related to the same range of age, education level and being young middle-aged.

**Figure 1:**
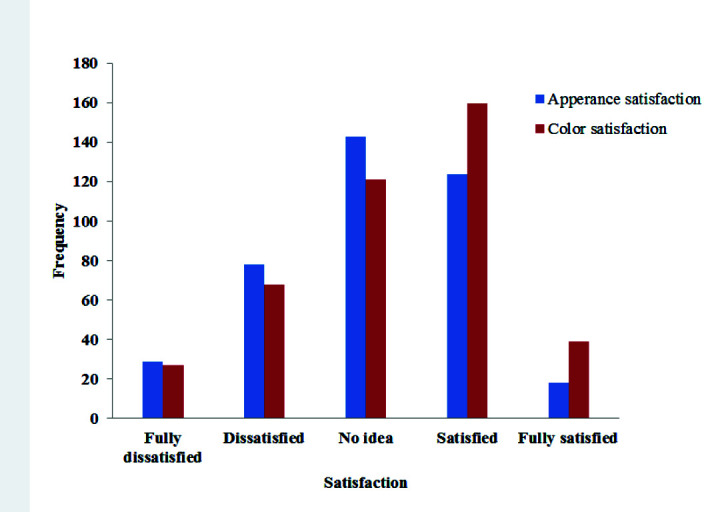
Frequency distribution of individuals according to tooth color and appearance satisfaction

In addition, 49.75% of the participants (Figure 1) were satisfied and quite satisfied with the appearance of their teeth. Various studies [ 31, 17, 26- 28 ] in different countries have reported different levels of satisfaction, including 42.2% in Malaysia [ 31 ], 57.3% in Turkey [ 17 ], 65 % in Palestine [ 26 ], 65.5 % in Jordan [ 27 ], and 76% in England (76%) [ 28 ]. Compared to our study, Yu *et al*. [ 24 ] showed, 53.6% of Chinese students were dissatisfied with the appearance of their teeth.

According to our study, there was no statistically significant relationship between dental appearance satisfaction with educational field, age, and gender, but Strajnić *et al*. [ 29] found that with increasing education level, satisfaction with the appearance of teeth was also increased. As such, in our study, five completely satisfied to completely dissatisfied question options were used to measure the degree of tooth color satisfaction. This investigation shows that 13.5% of participants had done bleaching, while Hatherell *et al*. [ 30] revealed that 28.44% of the students had done bleaching, which was more than our study. This study also indicates that 69% of participants tended to do bleaching similar to that of Hatherell *et al*. [ 30], whereas 66.37% of students in the United Kingdom wanted to do tooth bleaching (Table 3). The tendency to do bleaching in the Al-Zarea study [ 23] was, 80.9%, Akarslan *et al*. [ 17], 49% Tin-O-o *et al*. [ 31], 55.3%, Nomay [ 21], 77% and Subait 80% [ 19].

In the study of Maghaireh et al. [ 16], the most tendency for improving the appearance of teeth was tooth bleaching and it also was the most favorable treatment in Riyadh, London and Malaysia [ 19, 31- 32].

In our study, female students were more likely to do bleaching, although the difference was not statistically significant. This can be attributed to the studied population and methods of assessing the tooth color satisfaction. In addition, 58.25% of students had heard about bleaching, but female students had significantly more hearing.

**Figure 2:**
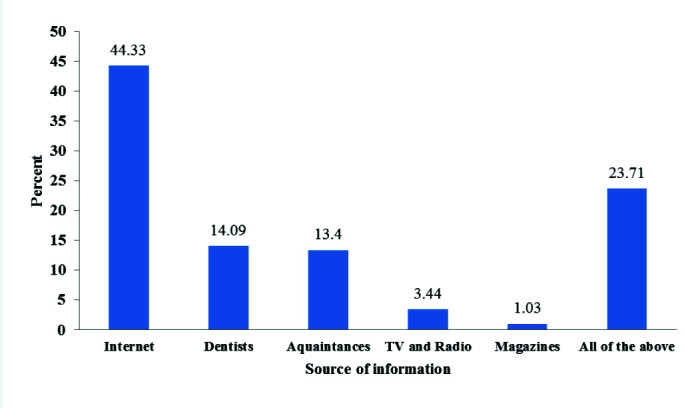
Frequency distribution of individuals according to source of information

The main source of information in 44.33% of participants was Internet and the next levels were dentists, friends, and acquaintances (Figure 2), which is somehow higher than the results (35.8 %) reported by Azodo *et al*. [ 14 ] in Nigeria. The reason for this difference may be due to greater access to the Internet in the current study.

Additionally, the most tendency was to do bleaching in the office (47.41%), thus dentists appear to be aware of the success rate of treatment and its limitations before any bleaching procedure.

The second method of bleaching was using bleach toothpaste (25.29%), although Wash *et al*. [ 33] and Joiner *et al*. [ 34] have shown that tooth discoloration may occur after regular use of toothpaste containing bleach material. Among our participants, only 1.4% of people tended to use bleach tape for bleaching.

Data analysis indicated that there was no significant difference between the age of the subjects and their knowledge of bleaching. This contrasts the results of Al-Nomay *et al*. [ 21] who studied different age groups, whereas our results were rather based on the similar age groups. 

There was no significant difference between participants' knowledge of dental bleaching in terms of gender and field of study. These results are not in line with Al-Nomay *et al*. study [ 21] that showed women were significantly more aware of the subject.

This study showed that 52.1% of students knew the difference between bleaching and SRP, even though our research had "no idea" option too (Table 4).

**Table4:** Frequency distribution of answers to the knowledge questions.

Question	Yes		No		No idea	
	Number	Percent	Number	Percent	Number	Percent
Does dental bleaching have any adverse effects?	104	25.9	23	5.7	267	66.6
Is dental bleaching safe for children and pregnant women?	28	7	77	19.2	288	71.8
Is there any difference between SRP and bleaching?	209	52.1	26	6.5	155	38.7
Is tooth bleaching a permanent treatment?	15	3.7	193	48.1	179	44.6
Is there any possibility for re-bleaching?	120	29.9	17	4.2	250	62.3
Does tooth bleaching have the ability for fading white spots?	51	12.7	51	12.7	285	71,1
Are crowns and laminates merely suitable for tooth whitening?	43	10.7	110	27,4	237	59,1
Do you know the degree of tooth whitening after bleaching?	Weakly whitened	Moderately whitened	Stronglywhitened		No idea	
	66 (16.5%)	45 (11.2%)	40 (10%)		237 (59.1%)	

In addition, 25.9% of the students answered yes to the question of side effects of bleaching, which is less than 46 % reported by Diklić *et al*. [ 35]. Regression analysis showed that the tooth color satisfaction increased the odds of tendency for doing bleaching to 1.87 times. This may be due to the age range of the participants, the uniformity of their education, and appropriate health behavior. However, tooth color dissatisfaction can influence people's decision to do bleaching.

Despite the results of this study, the most confounding factors which affect the tendency to do bleaching, include the treatment cost, the long multiple visits for in-office bleaching, and tooth sensitivity with nausea after each visit of tooth bleaching. In addition, the participants of our study were at the same age range and education, which could be effective on our results. Therefore, further investigations are needed concerning the knowledge and tendency of the public population to do dental bleaching.

## Conclusion

The present investigation highlights that 35.5% of the subjects were satisfied with the color, 49.75% with the appearance of the teeth. Moreover, 69% had a tendency to do bleaching and the knowledge level about bleaching and its alternative treatments was moderate.
